# Supplementary material for: Multiple molecular pathways to longevity with opposing gene expression programs defining distinct aging strategies in Caenorhabditis elegans
Source: eLife. 2026 Aug 20;15:RP112139. doi: 10.7554/eLife.112139 (PMC13493127; doi:10.7554/eLife.112139)
Supplement: Peer review history

# Editor report: eLife Assessment

**DOI:** 10.7554/eLife.112139.3.sa0

**Authors:** Scott F Leiser

**Affiliations:** University of Michigan United States

**Keywords:** Solid, Valuable

This **valuable** study presents a comparative analysis of the transcriptomic features underlying *C. elegans* longevity, providing insights into how different changes in gene expression can promote longevity. The authors present **solid** evidence with analysis and selected functional validation showing that some long-lived animals share common changes, while others appear to use opposing strategies. The datasets and analyses contained within and the user-friendly website developed will be of interest to researchers interested in complicated transcriptomic analyses and/or the biology of aging.

[Editors' note: this paper was reviewed by Review Commons.]

# Referee report: Reviewer #1 (Public review)

**DOI:** 10.7554/eLife.112139.3.sa1

[Editors' note: this version has been assessed by the Reviewing Editor without further input from the original reviewers.]

This manuscript by Rudich ZD et al. systematically profiled the transcriptomic changes in nine long-lived *C. elegans* mutants and presented a careful and informative comparative analysis of these aging-related changes. In addition to these valuable datasets and bioinformatics analyses, the authors performed a large-scale RNAi screen to assess the role of the differentially expressed genes (DEGs) in these mutants and identify several potential targets to promote healthy aging. Moreover, the authors have provided a user-friendly website to examine genes of interest in those longevity mutants from their datasets.

Strengths:

Compared to previous transcriptomic analyses of these mutants in different reports, this study minimized the technical variations and benefitted from the advances in RNA-Seq technology and bioinformatics tools. Therefore, it should provide a more consistent and comprehensive view of the molecular mechanisms underlying the longevity of these mutants. The datasets in this manuscript are valuable to other researchers in the biology of aging.

Weaknesses:

Meanwhile, since these mutants have been extensively studied, the advance of this study in unknown ageing mechanisms remains limited.

Comments on revised version.

The authors addressed the concerns successfully.

# Referee report: Reviewer #2 (Public review)

**DOI:** 10.7554/eLife.112139.3.sa2

Summary:

In the manuscript titled "Multiple Molecular Pathways to Longevity: Opposing Gene Expression Programs Define Distinct Aging Strategies", the authors investigated diverse genetic pathways that contribute to lifespan extension in *Caenorhabditis elegans* and aimed to identify shared and distinct molecular mechanisms among various longevity mutants. Through comprehensive RNA sequencing of different longevity mutants representing seven distinct pathways, the authors showed that these mutants cluster into three primary groups based on their gene expression profiles. This transcriptomic analysis revealed that while some longevity genes are commonly regulated across multiple pathways, others exhibit opposing expression patterns, suggesting that distinct molecular strategies can lead to increased lifespan. Specifically, they identified a set of 196 genes that are consistently upregulated in most longevity mutants, many of which are involved in innate immunity and stress defense. By performing RNAi-based screening, the authors further validated the functional roles of several candidates, including C08F11.7, ugt-62, and K05C4.9, supporting their contributions to longevity and stress resistance. The authors conclude that longevity is mediated through multiple molecular pathways and provide a public online tool to study these complex transcriptomic landscapes.

Significance:

This study provides a systematic, side-by-side transcriptomic comparison of nine genetically distinct long-lived *C. elegans* mutants, revealing that lifespan extension arises from both shared and opposing gene expression programs. By identifying three distinct longevity groups and demonstrating that key pathways can be modulated in opposite directions to achieve long life, the work challenges the notion of a single universal transcriptional signature of aging. Importantly, functional validation shows that select commonly regulated genes can directly modulate lifespan and stress resistance, highlighting actionable molecular targets for promoting healthy aging.

Comments on revised version:

The authors addressed my concerns successfully.

# Author comment: Author response

**DOI:** 10.7554/eLife.112139.3.sa3

**Authors:** Zenith D Rudich, Jiaxi Guan, Aura A Tamez Gonzalez, Grant F Booth, Sonja K Soo, Ulrich Anglas, Meeta Mistry, Megan M Senchuk, Jeremy Van Raamsdonk

**Affiliations:** McGill University Montreal Canada; McGill University Montreal Canada; McGill University Montreal Canada; McGill University Montreal Canada; McGill University Montreal Canada; McGill University Montreal Canada; Harvard School of Public Health Boston United States; Van Andel Research Institute Grand Rapids United States; McGill University Montreal Canada

The following is the authors’ response to the original reviews

**Reviewer #1 (Public review):**

In the revised manuscript, the authors have addressed most of my concerns. In the text of this manuscript, the authors should still include more discussion on why osm-5 and daf-2 are categorized into two different groups.

According to this suggestion, we have expanded our discussion to discuss why osm-5 and daf-2 worms fall into different longevity groups despite the fact that disruption of DAF-16 decreases both of the their lifespans. Please see lines 363-376.
